# Detection of germline variants with pathogenic potential in 48 patients with familial colorectal cancer by using whole exome sequencing

**DOI:** 10.1186/s12920-023-01562-3

**Published:** 2023-06-09

**Authors:** Ashish Kumar Singh, Bente Talseth-Palmer, Alexandre Xavier, Rodney J. Scott, Finn Drabløs, Wenche Sjursen

**Affiliations:** 1 Department of Medical Genetics, St. Olavs Hospital, Trondheim, Norway; 2 Department of Clinical and Molecular Medicine, Faculty of Medicine and Health Sciences, NTNU - Norwegian University of Science and Technology, Trondheim, Norway; 3 School of Biomedical Science and Pharmacy, Faculty of Health and Medicine, University of Newcastle and Hunter Medical Research Institute, Newcastle, Australia; 4 Møre and Romsdal Hospital Trust, Research Unit, Ålesund, Norway; 5 NSW Health Pathology, Newcastle, Australia

**Keywords:** Whole exome sequencing (WES), Colorectal cancer (CRC), Lynch syndrome (LS), Familial colorectal cancer Type X (FCCTX), Mismatch repair (MMR), Copy number variation (CNV), Variant annotation, Variant filtration

## Abstract

**Background:**

Hereditary genetic mutations causing predisposition to colorectal cancer are accountable for approximately 30% of all colorectal cancer cases. However, only a small fraction of these are high penetrant mutations occurring in DNA mismatch repair genes, causing one of several types of familial colorectal cancer (CRC) syndromes. Most of the mutations are low-penetrant variants, contributing to an increased risk of familial colorectal cancer, and they are often found in additional genes and pathways not previously associated with CRC. The aim of this study was to identify such variants, both high-penetrant and low-penetrant ones.

**Methods:**

We performed whole exome sequencing on constitutional DNA extracted from blood of 48 patients suspected of familial colorectal cancer and used multiple in silico prediction tools and available literature-based evidence to detect and investigate genetic variants.

**Results:**

We identified several causative and some potentially causative germline variants in genes known for their association with colorectal cancer. In addition, we identified several variants in genes not typically included in relevant gene panels for colorectal cancer, including *CFTR*, *PABPC1* and *TYRO3*, which may be associated with an increased risk for cancer.

**Conclusions:**

Identification of variants in additional genes that potentially can be associated with familial colorectal cancer indicates a larger genetic spectrum of this disease, not limited only to mismatch repair genes. Usage of multiple in silico tools based on different methods and combined through a consensus approach increases the sensitivity of predictions and narrows down a large list of variants to the ones that are most likely to be significant.

**Supplementary Information:**

The online version contains supplementary material available at 10.1186/s12920-023-01562-3.

## Background

Cancer is a leading cause of premature mortality in the population [1] with 19.3 million newly diagnosed cases and 10 million deaths worldwide in 2020 [2]. Colorectal cancer (CRC) ranks third in cancer incidence, but second with respect to cancer-related mortality [2]. Of all CRC cases, 30% are thought to have a familial component but only one third of these are associated with a hereditary condition [3] where high-penetrance pathogenic variants account for their genetic predisposition leading to several types of familial CRC syndromes. A well-known example is defects in DNA mismatch repair (MMR) genes (*MLH1, MSH2, MSH6* & *PMS2*) leading to Lynch syndrome (LS). In addition to the MMR genes, pathogenic mutations in other known high penetrant CRC predisposing genes are relevant. Mutations in *APC* can lead to both classic (FAP) and attenuated familial adenomatous polyposis (AFAP), while mutations in *MUTYH* (biallelic) can lead to MUTYH-associated polyposis (MAP), mutations in *NTHL1* (biallelic) can lead to familial adenomatous polyposis 3 (FAP3), and mutations in *POLE* and *POLD1* can lead to Polymerase proofreading-associated polyposis (PPAP) [4–7]. For the remaining 20% of familial CRC causal genetic factors for CRC predisposition remain to be revealed. For example familial colorectal cancer Type X (FCCTX), a rare inherited cancer-predisposing syndrome, is characterized by the fulfilment of the Amsterdam criteria for Lynch syndrome, but genetic causes are not yet clear [8]. Next generation sequencing (NGS) and genome-wide association studies (GWASs) have been used to discover the etiology of familial CRC by identifying novel candidate genes and causal variants which have not yet been linked to CRC [4, 9]. Additionally, whole exome sequencing (WES) has been used to identify bi-allelic and polygenic mutations in FAP, LS or familial CRC cases [5, 10–12]. Polygenic variation is also recognized as a potential cause of increased disease penetrance in Lynch syndrome [13].

DNA sequencing of protein coding regions enables the study of novel candidate genes and their potential role in cancer risk. The selection of candidate genes can be based on prioritization scores [14]. However, in addition to the actual protein coding regions, WES by capturing may also provide some information about other genomic regions. With expanded sequencing kits, WES can widen the targeted content to include additional regions beyond exons, towards 5’ untranslated regions (5’UTRs) to capture transcription factor binding sites (TFBSs) and upstream open reading frames (uORFs), and towards 3’UTRs to reveal microRNA binding sites associated with gene regulation [15, 16].

Genetic variants are often classified as single nucleotide (nt) variation (SNV) (1 nt), short insertion-deletion variation (indel) (up to 50 nt), and structural variation (SV) (larger than 50 nt) [17]. In this context, SVs include insertions, deletions, duplications, inversions, translocations or a combination of these, co-occurring in a single genome as described by Calvalho et al. [18]. Deletions and duplications of SVs [19, 20], known as copy number variants (CNVs), have been associated with disease and can contribute to a large fraction of the disease-causing variation [21]. WES has mainly been used to detect disease-causing SNV/indel variants [22]. However, with the use of recently developed in silico methods it is possible to identify also CNVs from WES data [23].

An essential step in NGS data analysis is the assessment of a variant’s effect on gene function and any causative association with disease. This is achieved by assigning annotations consisting of both theoretical pathogenicity scores calculated by prediction tools and experimental data extracted from various databases. Annotation tools can provide a diverse set of annotations in one place [24, 25], and these annotations can then be used to filter down large lists of variants to the most significant ones. It has been shown that functional studies are necessary to unequivocally establish the role of variants in disease risk, see for example [26], and functional studies such as in vitro assays, animal models, or studies on patient-derived samples can provide important insights into the molecular mechanisms underlying variant effects [27]. However, functional studies are often time-consuming, expensive, and technically challenging, and not all variants may be amenable to such analyses. Computational annotation, made as reliable as possible, will therefore always be essential.

In the present study, WES was performed on constitutional DNA extracted from blood of 48 patients with suspected familial colorectal cancer. Variant calling was undertaken to detect SNVs, indels and CNVs in all target regions of the exome. Consensus prediction based on multiple in silico tools and literature-based evidence was used to search for disease association of detected variants. We identified several potentially causative germline pathogenic variants in genes known to be associated with colorectal cancer. Additionally, several variants were identified in genes normally not included in gene panels for colorectal cancer, and these may be associated with an increased cancer risk.

## Methods

### Samples and study design

Germline DNA were extracted from blood samples from 48 Australian patients diagnosed with colorectal cancer fulfilling the Amsterdam-II Criteria, including 16 related pairs of individuals from 8 families while 32 individuals were unrelated individuals. Sanger sequencing performed previously detected no germline MMR mutations in the samples. Therefore, these patients are defined as FCCTX patients. Table 1 gives an overview of the 48 patients, including their and family members cancer types.

**Table 1:** Overview of 48 patients and their family’s cancer history

ID (Fid)	CRC: Age of detection and diagnosis	Other cancers and age of detection	Family cancer history: Numbers of affected members and cancer types
S.13 (F.1)	Y (50)	-	4 CRC, 1 stomach, 1 melanoma
S.46 (F.1)	Y (46)	Lung (51)	
S.34 (F.2)	Y (48)	-	3 CRC, 2 melanoma, 1 ovarian, 1 leukaemia, 1lung, 1 kidney, 4 breast, 1 uterine
S.36 (F.2)	N	Uterine (41)	
S.47 (F.3)	Y (34)	Melanoma (32)	5 CRC, 2 melanoma, 3 breast,1 uterine, 2 kidney, 1 lung, 1 oesophagus, 1 bladder
S.48 (F.3)	N	Renal (53)	
S.37 (F.4)	Y (38)	-	2 CRC, 1 stomach, 1 throat
S.41 (F.4)	Y (53)	-	
S.23 (F.5)	Y (51)	-	4 CRC
S.25 (F.5)	Y (52)	-	
S.44 (F.6)	Y (33)	Uterine (NA)	3 CRC
S.45 (F.6)	N	Uterine (59)	
S.03 (F.7)	N	Ovarian (78)	1 CRC, 1 ovarian, 2 breast, 1 stomach, 1 renal, 1 liver
S.04 (F.7)	Y (31)	-	
S.01 (F.8)	Y (64)	-	3 CRC, 1 breast, 1 kidney
S.02 (F.8)	Y (30 s)	Breast (30 & 70)	
S.31	Y (57)	-	9 CRC
S.42	Y (12)	CRC (29)	-
S.19	Y (21)	CRC (40), non-hodgkin lymphoma (42)	-
S.27	N	Ureter (60)	6 CRC
S.18	Y (70)	-	5 CRC, 1 stomach, 1 lung, 1 cervical, 1 polyps
S.43	Y (51)	CRC (64)	5 CRC
S.38	Y (45)	-	5 CRC, 1 acute lymphoblastic leukaemia, 1 hodgkin lymphoma, 1 brain
S.06	Y (60)	Kidney (60), bladder (53)	5 CRC, 1 prostate
S.07	Y (50)	-	5 CRC, 1 uterine
S.24	Y (50)	-	5 CRC, 1 stomach
S.05	Y (64)	-	5 CRC, 1 breast, 1 jaw
S.12	Y (60)	-	4 CRC
S.15	Y (42)	-	4 CRC
S.21	N	Pancreas (62)	4 CRC
S.32	Y (58)	Pancreatic (60)	4 CRC, 3 unknown
S.40	Y (66)	Stomach (NA), kidney (NA)	4 CRC, 4 stomach, 1 brain, 4 eye, 2 lung
S.28	Y (57)	-	4 CRC, 1 kidney
S.17	Y (72)	Kidney (71)	4 CRC
S.08	Y (48)	-	4 CRC, 1 endometrial, 1 throat
S.29	Y (48)	Uterine (56)	3 CRC, 1 abdomen
S.10	Y (53)	Kidney (59)	3 CRC, 2 bladder, 1 lymphoma, 1 brain, 3 melanoma, 1 thyroid, 2 breast, 1 unknown
S.11	Y (60)	Bone (32), breast (57)	3 CRC
S.14	Y (52)	Breast (52)	3 CRC
S.35	Y (36)	Bladder (35), uterine (35)	2 CRC, 4 breast, 2 throat, 2 non-Hodgkin lymphoma
S.30	Y (68)	Ureter (79)	3 CRC, 1 prostate
S.22	Y (54)	CRC (55)	2 CRC, 2 unknown
S.20	Y (55)	Endometrial (41), breast (51)	3 CRC, 3 renal, 1 endometrial, 1 melanoma
S.09	Y (65)	Uterine (50)	2 CRC, 1 breast
S.16	Y (48)	CRC (67)	2 CRC, 1 stomach
S.33	Y (63)	Endometrial (29)	1 CRC, 1 leukaemia, 1 melanoma, 1 endometrial, 3 unknown
S.26	Y (65)	Endometrial (50)	1 Stomach, 1 throat
S.39	Y (77)	Renal (51), uterine (55)	1 CRC, 1 brain, 1 unknown

Age is in years

*Abbreviations: ID* Patient ID, *Fid* Family ID, *CRC* Colorectal cancer, *NA* Age not available

### Whole exome sequencing (WES)

WES was performed on germline DNA from these 48 samples. Paired-end library preparation was performed using the Illumina Truseq exome capturing kit. DNA was sheared to ~ 150 bp using the Bioruptor Pico (Diagenode) followed by the recommended protocol using a single index. The final libraries were sequenced using an Illumina Nextseq 500 kit (Illumina), 150 cycles pair ended. Libraries were quantified using Qbit High Sensitivity D100 (Agilent) and were checked using either TapeStation on Bioanalyzer (Agilent) for quality and size.

### Variant calling and annotation

SNV/indel variant calling was performed on the dataset using a standardized BWA-Picard-GATK pipeline [22]. Joint annotation of variants was performed using the command-line based batch annotation software tool Ensembl variant effector prediction (VEP) [24] complimented with additional annotations from database dbNSFP [28, 29] used as plugin with VEP.

Detection of CNVs was performed using an in-house developed method [30] for detecting CNVs in targeted sequencing data.

### Variant prioritization

Prioritization steps were performed on the initial set of 125.686 variants detected from variant calling on 48 samples, using the command-line based tool filter_vep from the VEP toolkit. This was performed in three stages. In stage one, variants were selected based on their frequency in the population database gnomAD (v2.1) [14]. Variants that were not present in the database were assigned a frequency of zero and were also included in the selection process. In the second stage, variants were classified and prioritized based on their clinical significance assigned in the ClinVar database [31]. In the third and final stage, variants passing through the previous two stages were filtered based on pathogenicity estimation scores of selected tools. This included REVEL [32], CADD [33], ClinPred [34], M-CAP [35], VEST4 [36], MetaSVM [37], BayesDel [38] for missense, nonsense and start-loss prediction; SpliceAI [39] for splicing alteration prediction; and Loftee [14] for loss of function prediction.

**Fig. 1:**
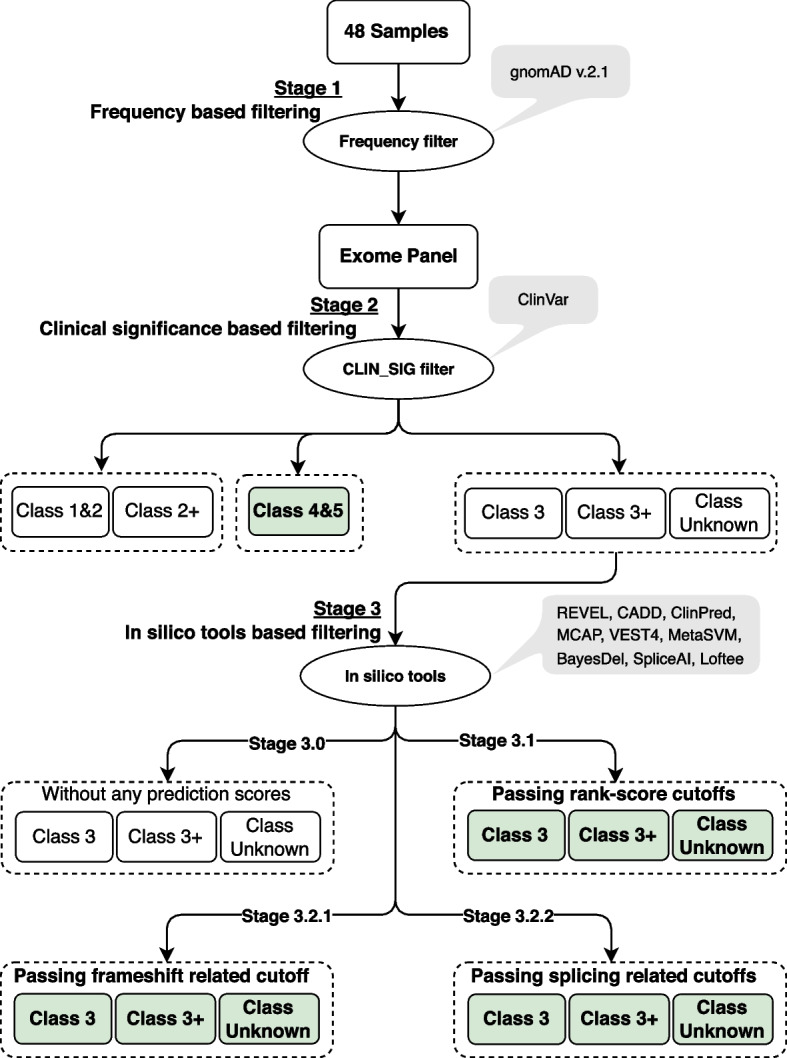
Variants filtering workflow, illustrating all filtering stages and their criteria. Variants in green boxes are the final output of filtration and are used for analysis

Selection of in silico prediction tools was based on ranking generated by our benchmarking study comparing the performance of 45 different pathogenicity prediction tools (see Supplementary file 1). We also took into consideration other benchmarking studies with similar goals [40–42]. Figure 1 shows workflow for these filtering steps and the outcome of each step. For detailed information about the various filtering steps, please see Supplementary file 2.

## Results

In all 48 samples, on average more than 99% of reads aligned to the reference genome GRCh37, with an average coverage depth of 92X. A total of 125.686 SNP/indel variants (for 25.664 genes) were identified in the 48 samples after the variant calling step. The three-stage filtering strategy detailed above (also displayed in Fig. 1) was applied to these variants, resulting in 346 variants (for 302 genes) with variants in different filtering categories. The average coverage depth at the variant position for these 346 variants was 110X, with a median of 91X and a maximum of 598X. However, there were four variants with a coverage depth of less than 10X at the variant position. The variants were assigned to different pathogenicity classes according to the ClinVar database. Table 2 displays a breakdown of these 346 variants into the number of variants for the different filtering steps. The full list of these variants is listed in Supplementary file 3.

**Table 2:** Number of variants as outputs from different filtering stages

**Variant calling**			125,686 variants (in 25,664 genes)					
**Stage 1**			22,626 variants (in 14,754 genes)					
**Stage 2**			**Class Unknown**	**Class 3**	**Class 3 + **	**Class 4&5**	**Class1&2**	**Class 2 + **
			20,541 variants (14,043 genes)	752 variants (849^**a**^ genes)	41 variants (37 genes)	**68** variants (91^**a**^ genes)	897 variants (1013^**a**^ genes)	340 variants (378^**a**^ genes)
**Stage 3**	**3.0**		14,467 variants (11,222 genes)	309 variants (382^**a**^ genes)	16 variant (5 genes)	No Stage 3 filtering in these classes (4&5, 1&2, 2 +)		
	**3.1**		**81** variants (78 genes)	**24** variants (24 genes)	**22** variants (21 genes)			
	**3.2**	**3.2.1**	**90** variants (85 genes)	0	**1** variant (1 gene)			
		**3.2.2**	**58** variants (43 genes)	**2** variants (2 genes)	0			

Filtering stages: Stage 1: Frequency (gnomAD) based filtering; Stage 2: Clinical significance (ClinVar) based filtering; Stage 3: Chosen in silico tools-based filtering, 3.0: Variants without any scores in chosen in silico tools, 3.1: Variants passing tool rank-scores cut-offs, 3.2.1: Variants passing splicing related filters, 3.2.2: Variants passing frameshift related filters

^a^Number of genes is higher than number of associated variants, due to multiple naming of some genes

To identify any associations of these variants with cancer all 302 genes were checked against three cancer-associated databases; COSMIC [43], OncoKB [44] and TSGene [45]. Only 38 of the 302 genes were listed in at least one of these databases. All these 38 genes are either known or have expected roles in cancer as oncogenes, tumor suppressor genes or fusion genes according to database classification. The list of the 38 genes with their respective roles in cancer are given in Supplementary Table S1. Of the 346 variants passing the filtering stages, only 45 variants are associated with any of these 38 genes. The variants associated with repetitive regions of these 38 genes were excluded. The average coverage depth at the variant position for these 45 variants was 94X, with a median of 79X and a maximum of 276X. The variant allele fraction (VAF) of these variants varied from minimum of 0.08 up to 1.65, with mean of 0.63.

Among these 45 variants, 14 are pathogenic/likely pathogenic, 6 are VUS, and 4 have conflicting interpretation between pathogenic/likely pathogenic and VUS according to ClinVar. The remaining 21 variants are not reported in ClinVar. Thirty-two of the 48 samples carried one or more of these 45 variants, the remaining 16 samples did not harbor any variant with a known cancer association, and hence lacked a clear link to an established cancer-associated variant. Table 3 lists the 32 samples and the associated 45 variants.

**Table 3:** List of 32 samples and associated 45 variants

ID (Fid)	gNomen, cNomen (pNomen), Existing variation	ClinVar	No. of samples
S.02 (F.8)	NM_022552.5(***DNMT3A***):c.2210 T > C (p.Leu737Pro)	NR	1
S.03 (F.7)	NM_000059.4(***BRCA2***):c.2808_2811del (p.Ala938Profs*21), rs80359351	P	2 (F.7)
	NM_006293.3(***TYRO3***):c.1660 + 1G > C (p.?), rs757748573	NR	3
	NM_001009944.3(***PKD1***):c.6605C > T (p.Ala2202Val), rs764264106	VUS	1
	NM_002894.3(***RBBP8***):c.298C > T (p.Arg100Trp), rs373804633	P	1
S.04 (F.7)	NM_000059.4(***BRCA2***):c.2808_2811del (p.Ala938Profs*21), rs80359351	P	2 (F.7)
	NM_003331.5(***TYK2***):c.1011 + 2 T > G (p.?), rs1463636749	NR	1
	NM_002568.4(***PABPC1***):c.739-1G > A (p.?), rs759516741	NR	7
S.08	NM_000492.4(***CFTR***):c.1392G > T (p.Lys464Asn), rs397508198	P	5
S.09	NM_007289.4(***MME***):c.467del (p.Pro156Leufs*14), rs749320057	P	1
	NM_001128840.3(***CACNA1D***):c.1750G > A (p.Val584Ile), rs773365038	VUS	1
S.11	NM_001349338.3(***FOXP1***):c.179A > G (p.Gln60Arg), rs374060287	LP	1
S.13 (F.1)	NM_006343.3(***MERTK***):c.1450G > A (p.Gly484Ser), rs527236084	VUS	1
	NM_002568.4(***PABPC1***):c.739-1G > A (p.?), rs759516741	NR	7
S.14	NM_005168.5(***RND3***):c.349-2A > T (p.?), rs1222374113	NR	1
S.15	NM_000088.4(***COL1A1***):c.4066C > A (p.Arg1356Ser), rs1341595487	VUS	1
	NM_002568.4(***PABPC1***):c.739-1G > A (p.?), rs759516741	NR	7
	NM_002568.4(***PABPC1***):c.388-1G > A (p.?), rs771446357	NR	1
S.16	NM_004431.5(***EPHA2***):c.2162G > A (p.Arg721Gln), rs116506614	CI	1
	NM_000492.4(***CFTR***):c.1392G > T (p.Lys464Asn), rs397508198	P	5
	NM_002568.4(***PABPC1***):c.739-1G > A (p.?), rs759516741	NR	7
S.17	NM_002911.4(***UPF1***):c.2474G > T (p.Ser825Ile)	NR	1
	NM_006941.4(***SOX10***):c.718A > C (p.Thr240Pro), rs1332625359	VUS	1
S.19	NM_006293.3(***TYRO3***):c.1660 + 1G > C (p.?), rs757748573	NR	3
	NM_000535.7(***PMS2***):c.614A > C (p.Gln205Pro), rs587779342	CI	2
	NM_000535.7(***PMS2***):c.1A > G (p.Met1Val), rs587779333	P/LP	2
	NM_002568.4(***PABPC1***):c.739-1G > A (p.?), rs759516741	NR	7
S.20	NM_002693.3(***POLG***):c.2209G > C (p.Gly737Arg), rs121918054	P/LP	1
	NM_022552.5(***DNMT3A***):c.1122 + 2 T > G (p.?), COSV53057339	NR	1
S.21	NM_000059.4(***BRCA2***):c.7977-1G > C (p.?), rs81002874	P	1
	NM_052839.4(***PANX2***):c.1479dup (p.Gly494Argfs*13)	NR	1
S.23 (F.5)	NM_000400.4(***ERCC2***):c.1480-1G > C (p.?), rs375284572	NR	1
	NM_000249.4(***MLH1***):c.514G > A (p.Glu172Lys), COSV51617106	NR	2 (F.5)
S.24	NM_033084.5(***FANCD2***):c.1588C > T (p.Arg530*), rs962867926	NR	1
	NM_004625.4(***WNT7A***):c.874C > T (p.Arg292Cys), rs104893835	P	1
	NM_006424.3(***SLC34A2***):c.1267G > A (p.Gly423Arg), rs769110830	NR	1
S.25 (F.5)	NM_000249.4(***MLH1***):c.514G > A (p.Glu172Lys), COSV51617106	NR	2 (F.5)
	NM_004168.4(***SDHA***):c.762_770 + 17del (p.Ala255_Gly257del), rs1041809852	P	1
S.27	NM_000492.4(***CFTR***):c.2723C > A (p.Thr908Asn), rs369521395	P	1
	NM_002568.4(***PABPC1***):c.739-1G > A (p.?), rs759516741	NR	7
S.29	NM_002568.4(***PABPC1***):c.739-1G > A (p.?), rs759516741	NR	7
S.30	NM_004963.4(***GUCY2C***):c.612-1G > A (p.?), rs763904634	NR	1
	NM_006293.3(***TYRO3***):c.308 + 1G > C (p.?), rs764446020	NR	1
	NM_000179.3(***MSH6***):c.3724_3726del (p.Arg1242del), rs63749942	P/LP	1
	NM_000492.4(***CFTR***):c.1392G > T (p.Lys464Asn), rs397508198	P	5
	NM_001001548.3(***CD36***):c.1202_1205del (p.Val401Glufs*4), rs769354931	CI	1
S.31	NM_006092.4(***NOD1***):c.689 T > G (p.Phe230Cys), CM1612670	NR	1
S.32	NM_007371.4(***BRD3***):c.71dup (p.Glu25Glyfs*51), rs768970491	NR	1
S.33	NM_002568.4(***PABPC1***):c.367G > T (p.Gly123Cys), rs755674364	NR	1
S.34 (F.2)	NM_001371290.1(***ZBTB7C***):c.402_403insC (p.Glu135Argfs*4)	NR	1
	NM_000492.4(***CFTR***):c.1392G > T (p.Lys464Asn), rs397508198	P	5
S.36 (F.2)	NM_145728.3(***SYNM***):c.2523del (p.His842Thrfs*47), COSV60376961	NR	1
	NM_000535.7(***PMS2***):c.614A > C (p.Gln205Pro), rs587779342	CI	2
	NM_000535.7(***PMS2***):c.1A > G (p.Met1Val), rs587779333	P/LP	2
S.37 (F.4)	NM_006293.3(***TYRO3***):c.1660 + 1G > C (p.?), rs757748573	NR	3
S.38	NM_024415.3(***DDX4***):c.673 + 2 T > C (p.?), rs201596382	NR	1
S.39	NM_000251.3(***MSH2***):c.2228C > G (p.Ser743*), rs63751155	P	1
S.43	NM_002335.4(***LRP5***):c.3562C > T (p.Arg1188Trp), rs141178995	P	1
S.44 (F.6)	NM_000264.5(***PTCH1***):c.104G > A (p.Arg35Gln), rs587778627	VUS	1
S.47 (F.3)	NM_000492.4(***CFTR***):c.1392G > T (p.Lys464Asn), rs397508198	P	5
S.48 (F.3)	NM_017563.5(***IL17RD***):c.392A > C (p.Lys131Thr), rs184758350	CI	1

*Abbreviations*: *ID* Patient ID, *Fid* Family ID, *LP* Likely pathogenic, *P* Pathogenic, *VUS* Uncertain significance, *LB* Likely benign, *NR* Not reported, *CI* Conflicting interpretations (P/LP; VUS)

Among these 38 genes, 7 are well known cancer genes with high impact towards cancer. These included *BRCA2, MLH1, MSH2, MSH6, PMS2, PTCH1* & *SDHA*. These 7 genes have 9 variants with pathogenicity classes 5, 4 or 3, occurring in 10 patients. Table 4 lists these genes and the associated variants.

**Table 4:** List of known cancer genes and associated variants

Gene	Linked to cancer	Variant	ACMG-AMP	ID (Fid)
*BRCA2* (NM_000059.4)	BrC	c.2808_2811del (p.Ala938Profs^*^21), rs80359351, VCV000009322.91	Class 5	S.03 (F.7)
				S.04 (F.7)
		c.7977-1G > C (p.?), rs81002874, VCV000038132.28	Class 5	S.21
*MLH1* (NM_000249.4)	CRC	c.514G > A (p.Glu172Lys), COSV51617106	Class 3	S.23 (F.5)
				S.25 (F.5)
*MSH2* (NM_000251.3)	CRC	c.2228C > G (p.Ser743^*^), rs63751155, VCV000090933.13	Class 5	S.39
*MSH6* (NM_000179.3)	CRC	c.3724_3726del (p.Arg1242del), rs63749942, VCV000089450.22	Class 4	S.30
*PMS2* (NM_000535.7)	CRC	c.614A > C (p.Gln205Pro), rs587779342, VCV000091361.24	Class 4	S.19
				S.36 (F.2)
		c.1A > G (p.Met1Val), rs587779333, VCV000091323.36	Class 5	S.19
				S.36 (F.2)
*PTCH1* (NM_000264.5)	GS	c.104G > A (p.Arg35Gln), rs587778627, VCV000135094.9	Class 3	S.44 (F.6)
*SDHA* (NM_004168.4)	PG	c.762_770 + 17del (p.Ala255_Gly257del), rs1041809852, VCV000412346.10	Class 3	S.25 (F.5)

*Abbreviations*: *ID* Patient ID, *Fid* Family ID, *CRC* Colorectal cancer, *BrC* Breast cancer, *GS* Gorlin syndrome, *PG* Paraganglioma

### Copy number variant calling

We performed CNVs calling step only for 88 known cancer genes. List of these genes is provided as Supplementary file 4. We detected 5 CNVs associated to 5 genes including 1 deletion and 4 duplications in 5 patients. More details are available as Supplementary Table S2.

## Discussion

This study uses a WES-based approach to identify the genetic causes of disease in FCCTX patients, where the majority of the patients had been diagnosed with CRC, and all fulfilling the AMS criteria. Most of the patients in this cohort were at the time of genetic testing pre-screened using denaturing high performance liquid chromatography (DHPLC) prior to Sanger sequencing. DHPLC is an inferior screening method that does miss some genetic variants and hence some samples were not further processed for Sanger Sequencing. The use of WES was therefore performed to identify relevant variants in additional cancer-associated genes, as well as the MMR genes. We identified significant variants in 38 genes known for cancer associations; this included 7 well established cancer genes with high cancer penetrance.

Four patients harbored pathogenic MMR variants, and therefore have a molecular diagnosis of Lynch syndrome. In addition, one suspicious VUS (*MLH1* c.514G > A) was detected in two members of family 5 and may also represent Lynch syndrome.

The *MLH1* variant c.514G > A (p.Glu172Lys) found in two patients from the same family (S.23 and S.25, family F.5), is a VUS with the potential to be pathogenic. Immunohistochemistry showed missing protein staining for *MLH1* and *PMS2* in tumors from both family members. The variant is not reported in gnomAD, and the REVEL score (0.876) indicates pathogenicity. Residue Glu172 is highly conserved and located in the ATPase domain of *MLH1*, within an α-helix structure. The switch from Glu to Lys results in a change from acidic to basic residue, which may disrupt the α-helix. In addition, this variant has been observed as a somatic change in three carcinomas (COSMIC database) of the breast, endometrium and large intestine.

*PMS2* has two mutations c.614A > C (p.Gln205Pro) and c.1A > G (p.Met1Val) classified as class 4 and class 5 respectively. Both were found in two unrelated patients, S.19 and S.36. These two variants have also been detected in one patient by a previous study [46]. For variant c.614A > C functional studies have demonstrated significantly higher repair efficiency than that of a pathogenic control, but 50% compromised when compared to wild type [47]. Biallelic defects in MMR genes are known as constitutive mismatch repair defect (CMMRD), and CMMRD patients often have more severe phenotypes than Lynch syndrome patients have. Previous studies have identified biallelic pathogenic *PMS2* mutations driven CMMRD leading to cancers in younger patients [48–50]. Patient S.19 was diagnosed with CRC at early age of 21 years and a second CRC at age of 40 years, whereas patient S.36 was diagnosed with uterine cancer at 41, and she did not develop CRC. We are not able to distinguish whether the two *PMS2* variants are biallelic or in cis (same allele) in these two patients. Gene *SDHA* has variant c.762_770 + 17del (p.Ala255_Gly257del) in patient S.25 (F.5), a deletion of three amino acids predicted to cause loss of a splice donor site (SpliceAI score:1). Loss of donor splice site is predicted to disrupt RNA splicing and culminate in either the absence or disruption of the protein product. As a tumor suppressor gene, *SDHA* is more likely to be associated with neuroendocrine related cancers, more commonly paragangliomas, with germline mutations accounting for 7.6% of patients with this cancer type [51].

Family 7 harbor a pathogenic *BRCA2* mutation c.2808_2811del causing hereditary breast and ovarian cancer. One of the two included family members had been diagnosed with ovarian cancer, while extended family members had breast and ovarian cancer, in addition to CRC. There has been a discussion whether pathogenic *BRCA1/2* variants are associated with CRC. However, a recent meta-analysis concluded that *BRCA1* and/or *BRCA2* mutation carriers are not at a higher risk of colorectal cancer [52].

The remaining 31 genes are associated with a variety of different roles including tumor suppressor genes, oncogenes, and fusion genes in various types of cancers (Supplementary Table S1). The identification of variants in these genes that have not previously been associated with familial CRC suggests a larger spectrum of genetic variants associated with this disease that is not limited to DNA mismatch repair genes or other known cancer-associated genes. Among these 31 candidate genes *CFTR*, *PABPC1*, and *TYRO3* have variants over-represented in this patient cohort.

We identified two pathogenic variants in gene *CFTR* (NM_000492.4); c.2723C > A (p.Thr908Asn) and c.1392G > T (p.Lys464Asn). Variant c.2723C > A is occurring in one patient whereas c.1392G > T is occurring in five patients, all five have CRC. That the pathogenic variant c.1392G > T (p.Lys464Asn) is over-represented in this cohort of cancer patients indicates that it could contribute to CRC development, but this needs further investigation. Previously, *CFTR* has primarily been associated with cystic fibrosis (CF) (a recessive disease), but has recently been categorized as a CRC risk gene [53]. This is seen as a result of CF patients surviving long enough to develop CRC. In addition, recent evidence indicates that low expression levels of *CFTR* is associated with a significant risk towards CRC [54].

Variants in the gene *PABPC1* (NM_002568.4) were found in nine patients, and the most frequently occurring variant (c.739-1G > A) was found in seven of these. The gene product of *PABPC1* is PABP-1, which is a poly(A) binding protein involved in several aspects of mRNA metabolism, including splicing of pre-mRNA, initiation of translation of mRNA, and mRNA decay [55]. *PABPC1* has been shown to be an oncogene that is upregulated in gastric carcinoma, where high expression predicts poor survival [56]. However, for esophageal cancer it has been shown that reduced expression of *PABPC1* correlates with tumor progression and poor prognosis after surgery [57], indicating a complex relationship between *PABPC1* expression levels and cancer. Recently *PABPC1* has been identified as a putative CRC driver gene in some patients [58]. Several domains have been identified in the protein, including four RNA recognition motif (RRM) domains, and a PAB C-terminal (PABC) domain that can bind interacting proteins [55]. One of the variants revealed in this study (rs759516741) has been classified as a splice acceptor, and the variant is located at position -1 relative to the start of exon 6 (NM_002568.4:c.739-1G > A). This exon is coding for residues 247 to 292 of the protein sequence, which overlaps partly with the third RRM domain (191–268). The variant may therefore affect the RRM 3 domain as well as domains further downstream, RRM 4 (294–370) and PABC (542–619). However, it is difficult to estimate how this may affect the function of PABP-1 and any processes where it is involved.

For *TYRO3* (NM_006293.3) two different variants in four patients were identified. The most frequent (c.1660 + 1G > C) was found in three patients. TYRO3 is a receptor tyrosine kinase of 890 residues, and signals are transduced into the cytoplasm when extracellular ligand binding induces dimerization and autophosphorylation of its intracellular domain [59]. TYRO3 acts as an oncogenic protein [60]. Overexpression has been observed in several cancers and is associated with a poor prognosis. Somatic mutations have also been observed, but without validation of their effect [59]. Regulation of *TYRO3* in CRC by specific non-coding RNA molecules has recently been documented [61, 62], and these studies also highlight the clear relationship between *TYRO3* overexpression and cancer. The most frequent variant in this dataset (rs757748573) has been classified as a splice donor variant. It is found at position + 1 relative to the end of exon 13 (NM_006293.3:c.1660 + 1G > C). The start of exon 14 corresponds to position 553 of the protein, which is in the intracellular domain (451–890). This means that the variant may affect signal transduction. However, whether that can give a similar metabolic effect as a general overexpression of *TYRO3* and activation of the protein is difficult to predict.

One among five detected CNVs, *RB1* (NM_000321.2) ex6.del, will most probably cause frameshift and affect the function of the gene. However, *RB1*, a tumor suppressor gene, often retains higher expression levels compared with adjacent normal tissue in CRC cells [63]. It is less likely that this CNV is associated to CRC and hence not significant. For the other four detected CNVs we could not establish any functional significance towards CRC.

Variant calling in whole exome regions identified 125.686 SNPs/indels in 25.664 genes. These numbers are too high for a detailed analysis of all cases; therefore, some filtering was needed to limit the number of variants for further analysis. To identify the variants that were most likely to have an effect on gene function, we applied a set of strict filtering criteria. Details of these criteria can be found in the Methods section. One of the initial filters used was based on variant frequency, with a cut-off of less than 0.1% in the gnomAD database. This cut-off is typically used for high-penetrance variants, while a cut-off of 1% is recommended for low-penetrance variants [64]. It is likely that this strict frequency threshold can result in the loss of some significant low-penetrance variants. However, this is not always the case. For example, previous studies have indicated that the *CFTR* gene contains disease-associated recessive variants with frequencies that are relatively low for such variants [53, 54].

In addition to frequency-based filters, we also applied stringent filters on prediction scores for variants using both individual tools as well as consensus predictions from multiple tools based on different prediction approaches. These filters resulted in a much smaller set of 346 variants that passed our criteria. Although the use of very strict filtering criteria increases the chance of detecting variants which are more likely to have a negative effect on gene function, it will also increase the risk of missing significant variants, causing a bias in the study. For example, a known (and likely pathogenic) variant NM_000251.3(*MSH2*): (p.Ala689Asp) was c.2066C > A identified in sample S.20 (not included in Table 4). Even though it had very high pathogenicity score by all seven prediction tools, it was filtered out by class unknown filters (*[T*_*2*_*-cutoff-0.99] || [T*_*7*_*-cutoff-0.8]*) by a very small margin (scored 0.78 rank score by CADD-raw). However, even a small adjustment towards less stringent filtering allowing this variant to pass, would also have increased the number of unknown variants after filtering by a minimum two-fold. The number of variants passing different combination filters based on rank-scores of 7 in silico tools is provided as Supplementary Table S3. Additionally, the *MSH2* c.2066C > A variant assigned as unknown (not reported) by VEP-based offline ClinVar annotation is a VUS according to the most recent online ClinVar records. Using this more recent ClinVar classification as VUS, i.e., a class 3 variant, it passes the class 3 filters (*[T*_*6*_*-cutoff-0.8] || [T*_*1*_*-cutoff-0.99]*). Hence, it is not only strict filtering but also the discrepancy between offline and online annotation records which may lead to a loss of significant variants during filtering. Another variant, *PTCH1* c.104G > A (p.Arg35Gln) a VUS passed the class 3 filters. Mutations in *PTCH1* can cause nevoid basal cell carcinoma syndrome (NBCCS) an autosomal dominant disorder commonly known as Gorlin syndrome [65]. This variant (c.104G > A) has very low rank-scores in all selected in silico tools except in M-CAP (rank-score of 0.99639) which let it pass the filtering. A stricter filtering may have removed this variant from the final list. These examples demonstrate the challenges of setting up filters for large datasets, e.g., from whole genome or exome sequencing. This may be a problem mainly in more explorative analysis where exome or genome wide data are analyzed and relatively stricter filtering is required in order to keep the number of variants at a manageable level. This will normally not be the case in diagnostic settings, where fewer and mainly well-characterized genes are screened. Hence a relatively smaller number of variants are used as input for filtering, and less strict filtering criteria may be applied. Variants passing filtering stage 3.0, i.e., variants without any prediction scores, were not included in final list. Class 3 & 3 + variants of this stage were briefly checked for any significance, but none were found. There is a large number of variants in class unknown passing filtering in this stage, which can be used in future studies.

Mean coverage of these 48 samples was 92X where 84.3% of all variants in these samples had coverage depth higher than 30X. But one of these samples (S.36) had low coverage depth of 9X. However, detected variants in this sample were known to variant databases ClinVar [31] and dbSNP [66] with enlisted phenotypic effects matching the patient’s phenotype. This supports our findings in this patient sample. One of the variants in this sample, *PMS2* c.614A > C (p.Gln205Pro), a class 4 variant has coverage depth of 7X. This variant has also been identified in one additional sample in the cohort.

VAFs of the 45 variants found in known cancer genes ranged from a minimum of 0.08 up to 1.65. Only one of these variants had VAFs below 0.1. The variant *PABPC1* c.739-1G > A (rs759516741), occurred in 7 patients in our study cohort and has a VAF of 0.08 (depth_ref,alt:35,3) in one of the 7 patients. It has also been submitted to the dbSNP database multiple times.

Validation of variants with an alternative technique (i.e., Sanger sequencing) could not be performed because most of the sample material has been exhausted. However, given the high accuracy of present day NGS-based detection of SNV/indel variants, additional validations are often not necessary [67]. To ensure the reliability of identified variants we used Alamut visual plus [68] to manually check each variant in the respective BAM files for correct identification.

In this cohort of 48 patients, 32 patients have variants in genes with known associations to cancer. Only 7 of these 32 patients have pathogenic or likely pathogenic variants (classified according to ACMG-AMP guidelines) in known cancer genes. Of these 7 patients, four harbor confirmed causative variants in MMR genes, thereby establishing a diagnosis of LS (Table 4). For 25 patients we detected significant variants in candidate genes with a potential to be associated with familial CRC. However, to fully establish the significance of these candidate genes and variants it is necessary to integrate genetic and clinical data with data from functional studies.

In the remaining 16 patients, we have not detected any significant variants passing our filtering criteria in known or candidate cancer-association genes. A possible explanation for missing variants in these 16 samples is the strict filtering criteria. Less strict filtering is one possible approach to identify significant variants in these samples. It is also possible to incorporate the combined effect of multiple variants as causative factor for disease susceptibility. The co-occurrence of multiple rare low-to-moderate risk alleles are likely to be associated with a complex genetic predisposition [69], as the combined effect of common low-risk loci is currently estimated to be up to 15% of the familial risk for cancer [70]. Polygenic risk score based models are one of the latest methods utilizing this approach [71]. Additionally, with exome sequencing deep intronic mis-splicing variants may be missed, and such variants also contribute towards cancer [72]. We also have not included variants in regulatory regions, e.g., variants in uORF (up-stream open reading frames) in our analysis, mainly because of very sparse annotation for such variants. This is mainly due to the fact that these regions are not commonly sequenced in targeted sequencing, hence annotation data for relevant tools (e.g., UTRannotator [73]) is very sparse. In addition to these factors, there are many more that can also lead to a missed molecular diagnosis [74] for these 16 samples, e.g., somatic mosaicism, epigenetic inheritance, technological limitations, non-genetic risk factors and the fact that the clinical diagnosis may be incorrect due to insufficient information.

## Conclusions

In this study we have used whole exome sequencing (WES) to identify germline variants with a pathogenic potential in patients with FCCTX. This provides an opportunity to identify important variants in the full set of genes, not limited to a predefined subset of genes from a gene panel. However, it also gives very large lists of variants where most are of uncertain significance. The use of consensus predictions for pathogenicity by combining multiple in silico tools based on different approaches helps in narrowing down the list to the variants that are most likely to affect gene function. Although a strict approach means that important variants may be missed out from detection, such filtering is still an essential step in the analysis of WES data. Our analysis identified possibly pathogenic variants in genes that have not previously been associated with familial CRC, such as *PABPC1* and *TYRO3*. This warrants further investigation to establish any potential role of these genes with respect to CRC. The results indicate that a larger spectrum of genes and genetic variants may be associated with this disease, not limited to the usual suspects like the DNA MMR genes.

## Supplementary Information

**Additional file 1. **Benchmarking study comparing the performance of 45 different pathogenicity prediction tools.

**Additional file 2. **Detailed information about the various filtering steps.

**Additional file 3. **The full list of 346 variants passing filtering steps.

**Additional file 4.** List of 88 known cancer genes used as targets for CNV calling.

**Additional file 5: Table S1.** List of 38 genes and their roles in Cancer. **Table S2.** The list of detected CNVs. **Table S3.** Number of variants passing different combinations of filters based on rank-scores of 7 in silico tools.

## Data Availability

The raw data of whole-exome sequencing of the patients in this study and the full list of variants called are not publicly available to protect participant confidentiality. All variants after filtering are given in the paper. Further information about the data and conditions for access can be provided by the corresponding author AKS (Ashish.Kumar.Singh3@stolav.no; ashish.k.singh@ntnu.no) and by NSW Health Pathology, Newcastle, Australia (https://www.pathology.health.nsw.gov.au/).

## Abbreviations

CRC: Colorectal cancer
MMR: Mismatch repair
NGS: Next generation sequencing
WES: Whole exome sequencing
TFBS: Transcription factor binding sites
SNV: Single nucleotide variation
Indel: Insertion-deletion variation
CNV: Copy number variant
LlS: Lynch-like syndrome
FCCTX: Familial colorectal cancer Type X
DHPLC: Denaturing high performance liquid chromatography
CMMRD: Constitutive mismatch repair defect
