# Supplementary material for: Detection of germline variants with pathogenic potential in 48 patients with familial colorectal cancer by using whole exome sequencing
Source: BMC Med Genomics. 2023 Jun 9;16:126. doi: 10.1186/s12920-023-01562-3 (PMC10257304; doi:10.1186/s12920-023-01562-3)
Supplement: Supplementary file 1 — Additional file 1. Benchmarking study comparing the performance of 45 different pathogenicity prediction tools. [file 12920_2023_1562_MOESM1_ESM.pdf]

This document is included as Supplementary Material for the paper "Detection of germline variants with pathogenic potential in 48 patients with familial colorectal cancer by using whole exome sequencing" by Ashish Kumar Singh, Bente Talseth-Palmer, Alexandre Xavier, Rodney J Scott, Finn Drabløs and Wenche Sjursen

Evaluation of methods for computational prediction of pathogenicity of missense variants

Ashish Kumar Singh[1,2,*], *Finn Drabløs*[1,*,+]

1. Department of Clinical and Molecular Medicine, Faculty of Medicine and Health Sciences, NTNU - Norwegian University of Science and Technology, Trondheim, Norway
2. Department of Medical Genetics, St. Olavs Hospital, Trondheim, Norway
* The authors contributed equally.
+ Corresponding author <finn.drablos@ntnu.no>

Abstract

Background: Several methods have been developed for predicting pathogenicity of missense variants in protein-coding regions of genes. Here we try to evaluate the performance of several such methods on a relevant exome-wide dataset of variants associated with colorectal cancer, to identify well-performing methods.

Results: Area Under Curve (AUC) of Receiver Operating Characteristic (ROC) curves for the prediction of pathogenicity of the missense variants found in the exome data compared to the ClinVar classification of the same variants (pathogenic or benign) was estimated. This was used to rank the performance of 45 different tools or pathogenicity scores. This was compared to the performance of these tools according to several previous benchmarking studies, and the overall performance was used to select the best-performing tools.

Conclusions: The seven tools with best overall performance were selected. These tools were ClinPred, VEST4, BayesDel-addAF, REVEL, CADD, M-CAP, and MetaSVM.

Keywords: Pathogenicity, Missense variants, Prediction, AUC, ROC.

Background

This project is part of a larger project on identifying possibly causative variants in whole-exome sequencing (WES) data from cancer patients. In general, the goal of this part of the project was to find a suitable set of computational tools for identifying pathogenic variants in exomes. This should be more than one tool, as no single tool has perfect sensitivity (*i.e.*, being able to find all relevant pathogenic variants) and selectivity (*i.e.*, being able to distinguish perfectly between pathogenic and benign variants). Not only because this is a challenging computational problem, but also because the distinction between pathogenic and benign can be unclear and may depend upon cancer type. It has been shown that using a consensus prediction based on a combination of tools (for example in the form of meta-predictors) may improve the overall performance [1], although also the opposite effect

has been observed [2]. But the performance of each individual tool should in any case be as good as possible. The goal has therefore been to identify suitable prediction methods for pathogenicity of variants for a specific dataset on colorectal cancer. However, although this was done with a specific dataset in mind, it is likely that the analysis may have a more general relevance.

A standard approach based on receiver operating characteristics was used for assessing the performance of relevant methods. Score values for several different tools were computed on a set of WES samples. ClinVar [3] classification of the variants was added, and the sample set was split into pathogenic and benign variants, based on the ClinVar classification, leaving out variants without a clear classification. The performance of a given tool at a given cutoff for the computed score value can then be estimated as the number of true positive (TP), true negative (TN), false positive (FP) and false negative (FN) classifications, which can be used to compute the true positive rate (sensitivity) and the false positive rate (1-specificity) at that cutoff. This can be computed across all score values as a Receiver Operating Characteristic (ROC) curve, and the overall performance of the method can be estimated by the Area Under Curve (AUC) for the ROC. An AUC close to 1.0 will indicate a near perfect classification performance of that tool, whereas an AUC close to 0.5 indicates that the performance of the classifier is similar to random classification.

Methods

Please see the main paper for details on the sequencing data that were used. Briefly, germline DNA was extracted from blood samples of 48 patients diagnosed with colorectal cancer fulfilling the Amsterdam-II Criteria for Lynch syndrome, consisting of 32 unrelated individuals, and 16 related individuals from 8 different families. Sanger sequencing detected no germline MMR mutations in these samples. Whole exome sequencing was performed on the 48 samples. SNP/indel variant calling was performed using a standardized BWA-Picard-GATK pipeline [4]. The variants were annotated with VEP, the Ensembl Variant Effect Predictor [5], using data from dbNSFP [6, 7]. Filtering of variants for further analysis was done with local scripts. ROC and AUC values were computed with the ROCR tool [8] in R (https://www.r-project.org/) version 3.6.3 (2020), and also correlations were computed in R.

Results

The initial input file consisted of 350.126 variants, including variants assigned to alternative transcripts of the same gene. This list was annotated with VEP and then filtered according to two main criteria.

Following the VEP annotation all prediction tools giving score values as a rank score were selected, in total 45 different methods or score sets, although several of these represent variants of the same basic prediction method (like BayesDel_addAF and BayesDel_noAF, representing the same method with and without allele frequencies). Then the number of annotated variants for each set of scores were counted, and two methods were excluded for further analysis (LINSIGHT and MutPred) because of low coverage. The coverage of the remaining methods varied from 34.165 (LRT_converted) to 46.240 (CADD_raw, DANN, GenoCanyon), although most methods had coverage >40.000. Then, only fully annotated variants (*i.e.*, variants annotated by all selected methods) were used.

The variants were also annotated according to ClinVar classification (CLIN_SIG in VEP output), and only variants that could be identified as either pathogenic or benign were used. All variants classified

as *pathogenic* or *likely_pathogenic* were counted as pathogenic. All variants classified as *benign* or *likely_benign* were counted as benign. All variants classified for example only as uncertain (*uncertain_significance*) or with conflicting classifications (*benign, pathogenic*) were excluded.

This gave a final dataset consisting of 961 variants, with 161 variants classified as pathogenic and 800 variants classified as benign. This dataset was then analyzed with ROCR in R.

The performance (given as AUC) for the 15 tools with best performance are given in Table 1. The full table is given as Supplementary Table S1. The tools indicated in red were later selected to be used in the main project, please see Discussion for details.

Table 1 – AUC for the 15 best-scoring methods

Method	AUC
ClinPred_rankscore	0.9312
VEST4_rankscore	0.8858
BayesDel_addAF_rankscore	0.8844
REVEL_rankscore	0.8799
Eigen-raw_coding_rankscore	0.8666
BayesDel_noAF_rankscore	0.8653
Eigen-PC-raw_coding_rankscore	0.8600
CADD_raw_rankscore	0.8547
M-CAP_rankscore	0.8540
CADD_raw_rankscore_hg19	0.8415
Polyphen2_HDIV_rankscore	0.8325
MutationAssessor_rankscore	0.8319
MetaSVM_rankscore	0.8243
Polyphen2_HVAR_rankscore	0.8227

Discussion

The results in Table 1 show that in particular ClinPred [9] has a very good performance on our dataset. This is hardly surprising, since ClinPred was developed by using both data and a strategy that is similar to what we have used in this benchmarking. However, to have a good basis for selecting a set of tools, data from several benchmarking studies were used.

Three quite recent benchmarking studies have included the ClinPred method. The study by Anderson & Lassman [10] was an extension of a previous benchmarking [11] and compared 37 different tools. Briefly, they made a data set of pathogenic variants from ClinVar, and terms on human phenotypic abnormalities from the Human Phenotype Ontology (HPO) resource [12] were associated with the relevant genes. They then used area under the precision-recall curve to estimate tool performance for discriminating between pathogenic and benign variants. Based on this they recommended mainly three methods: BayesDel_addAF (*i.e.*, with allele frequencies), CADD, and ClinPred.

The study by Borges *et al.* [13] compared 33 different methods. They used a large set of disease-causing variants of a specific gene (alpha-L-iduronidase, IDUA, involved in mucopolysaccharidosis type

I, MPS I) and used several statistical tests to evaluate how well each method could distinguish between deleterious and neutral variants. Based on this analysis the authors recommended in particular BayesDel (addAF and noAF), PON-P2 (genome and protein), and ClinPred.

Finally, the study by Gunning *et al.* [2] used two datasets, one 'open' set with data from ClinVar and gnomAD [14], and one 'clinically representative' dataset with variants identified through exome sequencing from diagnostics and research. They used this to evaluate a small number of methods using AUC for ROC curves, comparing three different meta-predictors (REVEL, GAVIN and ClinPred) against two commonly used *in silico* tools (SIFT and PolyPhen-2). They confirmed a good performance of in particular REVEL and ClinPred.

These studies confirm that ClinPred has a very good performance. However, there are also several other recent studies that can be relevant, with comparison or benchmarking of several methods. Suybeng *et al.* [15] used AUC scoring of more than 20 tools on a gold standard set of somatic single-nucleotide variants classified as oncogenic or neutral, and found the best performing tools to be CADD, Eigen, PolyPhen-2, PROVEAN, UMD-Predictor and REVEL.

Tian *et al.* [16] compared 7 predictors, using a high-quality consensus set of missense variants in clinically relevant genes which had been classified and reviewed by experts. Here REVEL and BayesDel showed the best performance.

Jaravine *et al.* [17] used machine-learning approaches to build ensemble or meta predictors consisting of several basic prediction methods, and by building these models in a stepwise manner (stacked ensembles) they could estimate how much each prediction method contributed to the overall performance of each ensemble. The best total performance was achieved with a distributed random forest (DRF), which showed that out of 39 different annotation scores (29 predictions, 9 conservation score and 1 indispensability score) tested on ClinVar-annotated variants from gnomAD [14], the best performance could be achieved with VEST4, M-CAP, CADD, MutPred, MVP and MetaLR, in that order.

Li *et al.* [18] used three different datasets for benchmarking, including ClinVar data, to compare 23 methods by using 12 different performance measures, including AUC. The methods included both function prediction methods, conservation methods and ensemble methods. It is difficult to extract overall recommendations from the results, but the AUC results for the ClinVar set showed particularly good performance for VEST3 and REVEL (AUC >0.9), but also very good performance for ensemble methods like CADD, Eigen, and MetaLR (AUC >0.87).

Chen *et al.* [19] used a slightly different approach where they focused on prediction of cancer driver mutations. They used five different datasets to compare 33 different methods. Based on AUC scores the methods like MetaSVM, MetaLR, M-CAP, and REVEL showed good performance on a dataset based on mutation clustering patterns, whereas PolyPhen2, PROVEAN, MetaLR, MutPred, REVEL, and VEST4 showed good performance on a dataset based on TP53 mutations.

Interestingly, our data and the benchmarking studies mentioned above show a quite consistent pattern with respect to identifying prediction methods with good performance. Based both on our own results and on benchmarking studies mentioned here several score methods could be relevant

for inclusion. However, to limit the number of methods it was also decided to leave out some methods that were very similar or highly correlated with already included methods, as it was assumed that such methods would provide limited additional information. Relevant examples are Eigen (correlation to CADD >0.92), MetaLR (similar to MetaSVM with correlation >0.94), and Polyphen2 (already included through most meta-methods). Also, MutationAssessor was left out, even though it had a quite good AUC score, because it was one of the methods with relatively low coverage with respect to the number of variants on which it had data (approximately 75% coverage compared to methods that were selected).

Based on the overall evaluation, 7 different methods were then selected to be used in a consensus-like approach in the main project; ClinPred [9], VEST4 [20], BayesDel-addAF [21], REVEL [22], CADD [23], M-CAP [24], and MetaSVM [25]. These methods are highlighted in Table 1, and the ROC curve for each of these methods is shown in Figure 1.

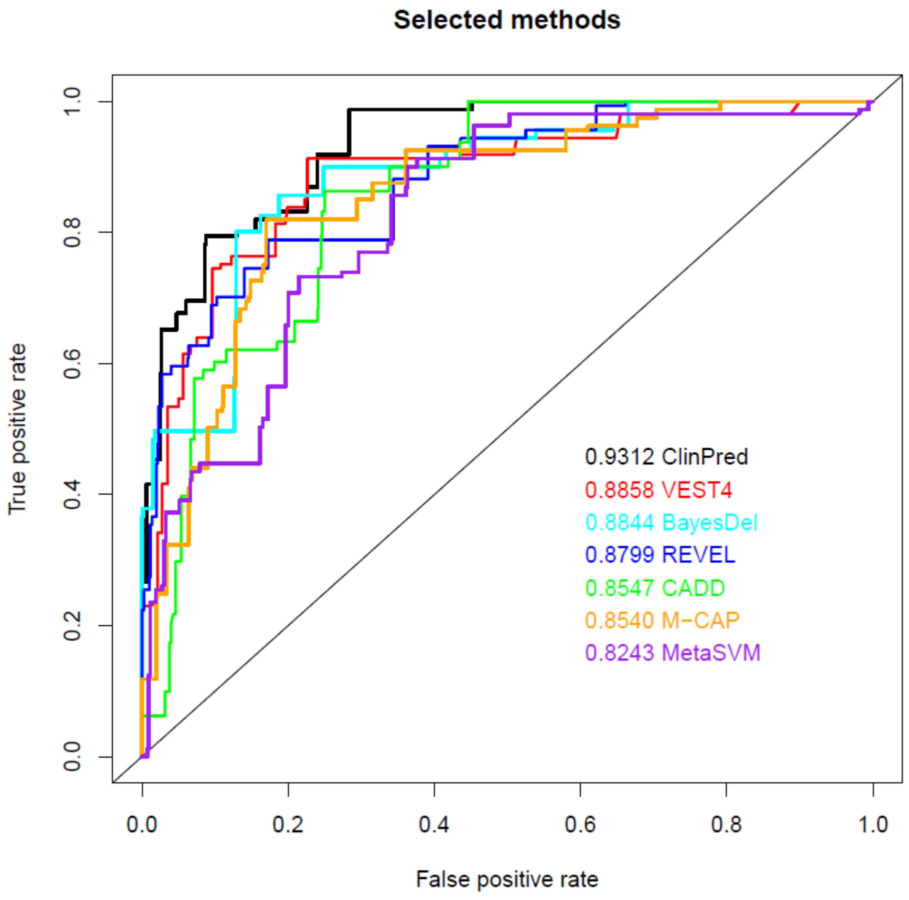

Figure 1 – ROC curve for selected methods

This analysis is included as supplementary material to the main paper, rather than a full, separate publication. It was mainly meant as a quick analysis to support the literature survey that already had been done, and to check whether this specific dataset behaved similarly to the more general benchmarking datasets used in published studies. There are several aspects of this study that certainly can be (and maybe should be) improved for this to be published as a proper benchmarking. It has not been attempted to do a selection of relevant transcripts, for example by using only

canonical transcripts. It can be argued that alternative transcripts in some cases may show different effects of specific variants, but the inclusion of several alternative transcripts can also lead to a bias towards genes with many know transcripts. It has not been attempted to make a more balanced dataset, with similar numbers of pathogenic and benign cases. This can also affect the analysis, although the effect here probably is small [9], at least partly because the AUC measure is supposed to be relatively robust for unbalanced datasets (see for example [10]). Finally, the interpretation of ClinVar classifications may be simplistic in cases with several alternative classifications. For example, a ClinVar classification as (*uncertain_significance, likely_pathogenic*) was selected to be counted as pathogenic, but this may not be a sufficiently reliable classification. However, the results from this analysis are similar to most of the benchmarking studies that we have compared it against, which seems to indicate that although the study was carried out in a relatively simplistic way (one might even say "quick-and-dirty"), the results seem to be quite robust and comparable to previous studies. Therefore, they provide a good basis for the selection of tools for the main project.

Conclusion

The study identified 7 different well-performing prediction methods for pathogenicity prediction of genetic variants. These 7 methods (ClinPred, VEST4, BayesDel-addAF, REVEL, CADD, M-CAP, and MetaSVM) were used in the main project, based on a consensus approach.

Author contributions

AKS processed sequencing data, identified variants, and annotated data with VEP. FD did the statistical analysis and wrote the first version of the manuscript. Both authors have approved the final version.

References

1. Gonzalez-Perez A, Lopez-Bigas N: **Improving the assessment of the outcome of nonsynonymous SNVs with a consensus deleteriousness score, Condel**. *Am J Hum Genet* 2011, **88**(4):440-449.
2. Gunning AC, Fryer V, Fasham J, Crosby AH, Ellard S, Baple EL, Wright CF: **Assessing performance of pathogenicity predictors using clinically relevant variant datasets**. *J Med Genet* 2021, **58**(8):547-555.
3. Landrum MJ, Lee JM, Benson M, Brown GR, Chao C, Chitipiralla S, Gu B, Hart J, Hoffman D, Jang W *et al*: **ClinVar: improving access to variant interpretations and supporting evidence**. *Nucleic Acids Res* 2018, **46**(D1):D1062-D1067.
4. Van der Auwera GA, Carneiro MO, Hartl C, Poplin R, Del Angel G, Levy-Moonshine A, Jordan T, Shakir K, Roazen D, Thibault J *et al*: **From FastQ data to high confidence variant calls: the Genome Analysis Toolkit best practices pipeline**. *Curr Protoc Bioinformatics* 2013, **43**:11 10 11-11 10 33.
5. McLaren W, Gil L, Hunt SE, Riat HS, Ritchie GR, Thormann A, Flicek P, Cunningham F: **The Ensembl Variant Effect Predictor**. *Genome Biol* 2016, **17**(1):122.
6. Liu X, Jian X, Boerwinkle E: **dbNSFP: a lightweight database of human nonsynonymous SNPs and their functional predictions**. *Hum Mutat* 2011, **32**(8):894-899.
7. Liu X, Li C, Mou C, Dong Y, Tu Y: **dbNSFP v4: a comprehensive database of transcript-specific functional predictions and annotations for human nonsynonymous and splice-site SNVs**. *Genome Med* 2020, **12**(1):103.
8. Sing T, Sander O, Beerenwinkel N, Lengauer T: **ROCR: visualizing classifier performance in R**. *Bioinformatics* 2005, **21**(20):3940-3941.
9. Alirezaie N, Kernohan KD, Hartley T, Majewski J, Hocking TD: **ClinPred: Prediction Tool to Identify Disease-Relevant Nonsynonymous Single-Nucleotide Variants**. *Am J Hum Genet* 2018, **103**(4):474-483.

10. Anderson D, Lassmann T: **An expanded phenotype centric benchmark of variant prioritisation tools**. *Hum Mutat* 2022.
11. Anderson D, Lassmann T: **A phenotype centric benchmark of variant prioritisation tools**. *NPJ Genom Med* 2018, **3**:5.
12. Kohler S, Doelken SC, Mungall CJ, Bauer S, Firth HV, Bailleul-Forestier I, Black GC, Brown DL, Brudno M, Campbell J *et al*: **The Human Phenotype Ontology project: linking molecular biology and disease through phenotype data**. *Nucleic Acids Res* 2014, **42**(Database issue):D966-974.
13. Borges P, Pasqualim G, Matte U: **Which Is the Best In Silico Program for the Missense Variations in IDUA Gene? A Comparison of 33 Programs Plus a Conservation Score and Evaluation of 586 Missense Variants**. *Front Mol Biosci* 2021, **8**:752797.
14. Karczewski KJ, Francioli LC, Tiao G, Cummings BB, Alfoldi J, Wang Q, Collins RL, Laricchia KM, Ganna A, Birnbaum DP *et al*: **The mutational constraint spectrum quantified from variation in 141,456 humans**. *Nature* 2020, **581**(7809):434-443.
15. Suybeng V, Koeppel F, Harle A, Rouleau E: **Comparison of Pathogenicity Prediction Tools on Somatic Variants**. *J Mol Diagn* 2020, **22**(12):1383-1392.
16. Tian Y, Pesaran T, Chamberlin A, Fenwick RB, Li S, Gau CL, Chao EC, Lu HM, Black MH, Qian D: **REVEL and BayesDel outperform other in silico meta-predictors for clinical variant classification**. *Sci Rep* 2019, **9**(1):12752.
17. Jaravine V, Balmford J, Metzger P, Boerries M, Binder H, Boeker M: **Annotation of Human Exome Gene Variants with Consensus Pathogenicity**. *Genes (Basel)* 2020, **11**(9).
18. Li J, Zhao T, Zhang Y, Zhang K, Shi L, Chen Y, Wang X, Sun Z: **Performance evaluation of pathogenicity-computation methods for missense variants**. *Nucleic Acids Res* 2018, **46**(15):7793-7804.
19. Chen H, Li J, Wang Y, Ng PK, Tsang YH, Shaw KR, Mills GB, Liang H: **Comprehensive assessment of computational algorithms in predicting cancer driver mutations**. *Genome Biol* 2020, **21**(1):43.
20. Carter H, Douville C, Stenson PD, Cooper DN, Karchin R: **Identifying Mendelian disease genes with the variant effect scoring tool**. *BMC Genomics* 2013, **14 Suppl 3**:S3.
21. Feng BJ: **PERCH: A Unified Framework for Disease Gene Prioritization**. *Hum Mutat* 2017, **38**(3):243-251.
22. Ioannidis NM, Rothstein JH, Pejaver V, Middha S, McDonnell SK, Baheti S, Musolf A, Li Q, Holzinger E, Karyadi D *et al*: **REVEL: An Ensemble Method for Predicting the Pathogenicity of Rare Missense Variants**. *Am J Hum Genet* 2016, **99**(4):877-885.
23. Kircher M, Witten DM, Jain P, O'Roak BJ, Cooper GM, Shendure J: **A general framework for estimating the relative pathogenicity of human genetic variants**. *Nat Genet* 2014, **46**(3):310-315.
24. Jagadeesh KA, Wenger AM, Berger MJ, Guturu H, Stenson PD, Cooper DN, Bernstein JA, Bejerano G: **M-CAP eliminates a majority of variants of uncertain significance in clinical exomes at high sensitivity**. *Nat Genet* 2016, **48**(12):1581-1586.
25. Dong C, Wei P, Jian X, Gibbs R, Boerwinkle E, Wang K, Liu X: **Comparison and integration of deleteriousness prediction methods for nonsynonymous SNVs in whole exome sequencing studies**. *Hum Mol Genet* 2015, **24**(8):2125-2137.

Supplementary

Supplementary Table S1 – AUC for all score methods

Method	AUC
ClinPred_rankscore	0.9312
VEST4_rankscore	0.8858
BayesDel_addAF_rankscore	0.8844
REVEL_rankscore	0.8799
Eigen-raw_coding_rankscore	0.8666
BayesDel_noAF_rankscore	0.8653
Eigen-PC-raw_coding_rankscore	0.8600
CADD_raw_rankscore	0.8547
M-CAP_rankscore	0.8540
CADD_raw_rankscore_hg19	0.8415
Polyphen2_HDIV_rankscore	0.8325
MutationAssessor_rankscore	0.8319
MetaSVM_rankscore	0.8243
Polyphen2_HVAR_rankscore	0.8227
SIFT4G_converted_rankscore	0.8131
MetaLR_rankscore	0.7890
fathmm-XF_coding_rankscore	0.7863
SIFT_converted_rankscore	0.7851
fathmm-MKL_coding_rankscore	0.7807
PROVEAN_converted_rankscore	0.7723
phyloP100way_vertebrate_rankscore	0.7644
phastCons100way_vertebrate_rankscore	0.7525
MVP_rankscore	0.7522
DANN_rankscore	0.7443
DEOGEN2_rankscore	0.7411
LIST-S2_rankscore	0.7312
GERP++_RS_rankscore	0.7213
LRT_converted_rankscore	0.7174
MutationTaster_converted_rankscore	0.7125
SiPhy_29way_logOdds_rankscore	0.6858
phastCons30way_mammalian_rankscore	0.6837
PrimateAI_rankscore	0.6727
FATHMM_converted_rankscore	0.6575
MPC_rankscore	0.6255
phyloP30way_mammalian_rankscore	0.6229
GenoCanyon_rankscore	0.6214
phastCons17way_primate_rankscore	0.6085
phyloP17way_primate_rankscore	0.5850
H1-hESC_fitCons_rankscore	0.4922
bStatistic_converted_rankscore	0.4907
integrated_fitCons_rankscore	0.4639
HUVEC_fitCons_rankscore	0.4545
GM12878_fitCons_rankscore	0.4239
