# Supplementary material for: Detection of germline variants with pathogenic potential in 48 patients with familial colorectal cancer by using whole exome sequencing
Source: BMC Med Genomics. 2023 Jun 9;16:126. doi: 10.1186/s12920-023-01562-3 (PMC10257304; doi:10.1186/s12920-023-01562-3)
Supplement: Supplementary file 2 — Additional file 2. Detailed information about the various filtering steps. [file 12920_2023_1562_MOESM2_ESM.pdf]

Variant prioritization

Prioritization steps were performed on initial set of 125686 variants detected via variant calling on 48 samples. Variant occurrences among global population, clinical significance based on already assigned predictions and pathogenicity estimation scores of selective tools (among full list of annotation tools) were taken in account. The selected tools were as follows:

- Database for calculation of population frequency: gnomAD version r2.1 (1)
- Database for estimation of clinical significance: ClinVar (2)
- *In silico* tools for pathogenicity prediction:
    - Missence, Nonsence and Start-loss prediction: REVEL (3), CADD (4), ClinPred (5), M-CAP (6), VEST4 (7), MetaSVM (8), BayesDel (9)
    - Splicing alteration prediction: SpliceAI (10)
    - Loss of function prediction: Loftee (1)

Selection of *in silico* prediction tools was based on ranking generated by our benchmarking study comparing performance of presently available 45 pathogenicity prediction tools (See supplementary document Sup1). Additionally we took inspiration from other benchmark studies with similar goals (11–13).

Variant filtration was performed using command-line based tool filter_vep form VEP toolkit. Filtering criteria have been explained in following stages:

1. First stage filtering was based on variant frequencies, where frequency database gnomAD (V2.1 i.e., only exome based frequency) was used to filter out variants with frequency higher then 0.001(gnomAD_AF < 0.001). Variants with no frequency (hit) in gnomAD database were considered with frequency of value 0, and have passed this filtering stage.
2. Second stage filtering was based on estimated clinical significance of variants provided by ClinVar database. Variants passing stage1 filtering were firstly categorized in below mentioned 6 different categories based on their clinical significance. Variants in Class1&2 and Class2+ where considered insignificant and were discarded after this stage. Variants in Class4&5 were manually curated after this stage. Remaining variants in categories Class3, Class3+ and ClassUnknown were further filtered in next stages based on different criteria.
    2.1. Class1&2: [benign OR likely benign]
    2.2. Class2+: [benign OR likely benign] AND [uncertain significance OR Conflicting interpretation]

2.3. Class3: [uncertain significance OR Conflicting interpretation]

2.4. Class3+: [pathogenic] AND [uncertain significance]

2.5. Class4&5: [pathogenic OR likely pathogenic]

2.6. Class Unknown: No Information in clinvar

3. Third stage filtering was based on predictions from 9 selected *in silico* tools for pathogenicity prediction. These tools were used in following three different ways to make filters. (Variants without any score (Stage 3.0) were not further analysed)

3.1. In stage 3.1, filters were made based on predictions from seven of these nine tools i.e., REVEL, CADD, BayesDel, ClinPred, M-CAP, MetaSVM and VEST4. These tools were used for predicting effects of missense, nonsense and start-loss related variants. Combinatorial approach was used to test the filter effects ahead of applying the filters, where all possible combinations of these seven tools with different rank-score cut-offs were tested on this filtration stage. Output of this analysis is provided as supplementary document (See supplementary table S4). Filters selected among those are described below.

3.1.1. Filter for Class3+: Variants with rank-score higher than or equal to 0.8 in at least one of these seven tools.

3.1.2. Filter for Class3: (Variants with rank-score higher than or equal to 0.99 in at least one of these seven tools) AND (Variants with rank-score higher than or equal to 0.8 in at least six on these seven tools).

3.1.3. Filter for ClassUnknown: (Variants with rank-score higher than or equal to 0.99 in at least two of these seven tools) AND (Variants with rank-score higher than or equal to 0.8 in all of these seven tools).

3.2. In stage 3.2, filtering was done to detect frameshift related and splicing-alteration variants.

3.2.1. Filter for frameshift variant: variants with consequence for frameshift and high confidence for loss-of-function from Loftee tool based annotation.

3.2.2. Filter for splicing-alteration: Variants with delta-score higher than or equal to 0.9 for any of four changes (i.e., acceptor-gain, acceptor-loss, donor-gain and donor-loss) and high confidence for loss-of-function from Loftee tool based annotation.

References:

1. Karczewski KJ, Francioli LC, Tiao G, Cummings BB, Alföldi J, Wang Q, et al. The mutational constraint spectrum quantified from variation in 141,456 humans. Nat 2020 5817809. 2020 May;581(7809):434–43.
2. Landrum MJ, Lee JM, Benson M, Brown GR, Chao C, Chitipiralla S, et al. ClinVar: improving access to variant interpretations and supporting evidence. Nucleic Acids Res. 2018 Jan;46(D1):D1062–7.
3. Ioannidis NM, Rothstein JH, Pejaver V, Middha S, McDonnell SK, Baheti S, et al. REVEL: An Ensemble Method for Predicting the Pathogenicity of Rare Missense Variants. Am J Hum Genet. 2016 Oct;99(4):877–85.
4. Rentzsch P, Witten D, Cooper GM, Shendure J, Kircher M. CADD: predicting the deleteriousness of variants throughout the human genome. Nucleic Acids Res. 2019 Jan;47(D1):D886–94.
5. Alirezaie N, Kernohan KD, Hartley T, Majewski J, Hocking TD. ClinPred: Prediction Tool to Identify Disease-Relevant Nonsynonymous Single-Nucleotide Variants. Am J Hum Genet. 2018 Oct;103(4):474–83.
6. Jagadeesh KA, Wenger AM, Berger MJ, Guturu H, Stenson PD, Cooper DN, et al. M-CAP eliminates a majority of variants of uncertain significance in clinical exomes at high sensitivity. Nat Genet. 2016;48(12):1581–6.
7. Madej T, Lanczycki CJ, Zhang D, Thiessen PA, Geer RC, Marchler-Bauer A, et al. MMDB and VAST+: tracking structural similarities between macromolecular complexes. Nucleic Acids Res. 2014 Jan;42(D1):D297–303.
8. Kim S, Jhong J-H, Lee J, Koo J-Y. Meta-analytic support vector machine for integrating multiple omics data. BioData Min. 2017;10(1):2.
9. Feng B-J. PERCH: A Unified Framework for Disease Gene Prioritization. Hum Mutat. 2017 Mar;38(3):243–51.
10. Jaganathan K, Kyriazopoulou Panagiotopoulou S, McRae JF, Darbandi SF, Knowles D, Li YI, et al. Predicting Splicing from Primary Sequence with Deep Learning. Cell. 2019 Jan;176(3):535-548.e24.
11. Anderson D, Lassmann T. An expanded phenotype centric benchmark of variant prioritisation tools. Hum Mutat. 2022 Feb;n/a(n/a).
12. Borges P, Pasqualim G, Matte U. Which Is the Best In Silico Program for the Missense

Variations in IDUA Gene? A Comparison of 33 Programs Plus a Conservation Score and Evaluation of 586 Missense Variants [Internet]. Vol. 8, Frontiers in Molecular Biosciences . 2021.

13. Gunning AC, Fryer V, Fasham J, Crosby AH, Ellard S, Baple EL, et al. Assessing performance of pathogenicity predictors using clinically relevant variant datasets. J Med Genet. 2021 Aug;58(8):547 LP – 555.
