# Supplementary material for: Detection of germline variants with pathogenic potential in 48 patients with familial colorectal cancer by using whole exome sequencing
Source: BMC Med Genomics. 2023 Jun 9;16:126. doi: 10.1186/s12920-023-01562-3 (PMC10257304; doi:10.1186/s12920-023-01562-3)
Supplement: Supplementary file 5 — Additional file 5: Table S1. List of 38 genes and their roles in Cancer. Table S2. The list of detected CNVs. Table S3. Number of variants passing different combinations of filters based on rank-scores of 7 in silico tools. [file 12920_2023_1562_MOESM5_ESM.pdf]

Table S1: List of 38 genes and their roles in Cancer (as per COSMIC, OncoKB, and TSGene databases)

Gene	Role in Cancer	Database
BRCA2	TSG	cosmic; OncoKB; TSG-db
BRD3	oncogene; fusion; In Sanger Cancer Gene Census	cosmic; OncoKB
CACNA1D	oncogene; In Sanger Cancer Gene Census	cosmic; OncoKB
CD36	In cancer panels (FOUNDATION ONE HEME)	OncoKB
CFTR	TSG	TSG-db
COL1A1	fusion; In cancer panels (FOUNDATION ONE HEME)	cosmic; OncoKB
DDX4	Oncogene	OncoKB
DNMT3A	TSG	cosmic; OncoKB; TSG-db
EPHA2	TSG	TSG-db
ERCC2	TSG	cosmic; OncoKB
FANCD2	TSG	cosmic; OncoKB
FOXP1	oncogene; fusion; TSG	cosmic; OncoKB; TSG-db
GUCY2C	TSG	TSG-db
IL17RD	TSG	TSG-db
LRP5	oncogene; TSG	OncoKB
MERTK	In cancer panels (FOUNDATION ONE)	OncoKB
MLH1	TSG	cosmic; OncoKB; TSG-db
MME	TSG	TSG-db
MSH2	TSG	cosmic; OncoKB; TSG-db
MSH6	TSG	cosmic; OncoKB
NOD1	In cancer panels (FOUNDATION ONE HEME)	OncoKB
PABPC1	oncogene; TSG	cosmic
PANX2	TSG	TSG-db
PKD1	TSG	TSG-db
PMS2	TSG	cosmic; OncoKB
POLG	TSG; Cancer gene (OncoKB assigned)	cosmic; OncoKB
PTCH1	TSG	cosmic; OncoKB; TSG-db
RBBP8	TSG	TSG-db
RND3	TSG	TSG-db
SDHA	TSG	cosmic; OncoKB; TSG-db
SLC34A2	TSG; fusion; In cancer panels (FOUNDATION ONE)	cosmic; OncoKB
SOX10	In cancer panels (FOUNDATION ONE HEME)	OncoKB
SYNM	TSG	TSG-db
TYK2	Oncogene	OncoKB
TYRO3	In cancer panels (FOUNDATION ONE)	OncoKB
UPF1	In cancer panels (MSK-IMPACT;MSK-HEME)	OncoKB
WNT7A	TSG	TSG-db
ZBTB7C	TSG	TSG-db

Table S2: The list of detected CNVs.

Sample ID	Gene: exon	Genomic position	CNV Type
S.01	RB1: exon6	chr13:48923092-48923159	Deletion
S.03	BARD1: exon5	chr2:215633956-215634036	Duplication
S.14	BLM: whole gene	chr15:91290623-91358509	Duplication
S.19	POLE: exon12	chr12:133251984-133252103	Duplication
S.34	PTCH2: exon22	chr1:45288087-45288341	Duplication

Table S3: Number of variants passing different combinations filters based on rank-scores of 7 in silico tools (CADD, ClinPred, M-CAP, BayesDel-addAF, MetaSVM, REVEL, VEST4)

**Class4-5**	Number of variants **(**and associated genes**)** passing filter: **[**rank-score>=Cutoff in at least **N** of **7** meta-tools**]**						
Total: 68 (91)	**N=1**	**N=2**	**N=3**	**N=4**	**N=5**	**N=6**	**N=7**
Cutoff>=0.8	49 (46)	45 (43)	32 (31)	24 (23)	14 (14)	8 (8)	4 (4)
Cutoff>=0.85	46 (45)	39 (38)	28 (28)	14 (14)	9 (9)	4 (4)	3 (3)
Cutoff>=0.9	41 (40)	33 (32)	18 (17)	10 (10)	5 (5)	3 (3)	0
Cutoff>=0.95	34 (33)	20 (20)	12 (12)	7 (7)	2 (2)	1 (1)	0
Cutoff>=0.99	12 (11)	3 (3)	2 (2)	0	0	0	0
**Class3+**	Number of variants **(**and associated genes**)** passing filter: **[**rank-score>=Cutoff in at least **N** of **7** meta-tools**]**						
Total: 41 (37)	**N=1**	**N=2**	**N=3**	**N=4**	**N=5**	**N=6**	**N=7**
Cutoff>=0.8	**22 (21)**	20 (19)	15 (14)	14 (13)	9 (8)	4 (4)	1 (1)
Cutoff>=0.85	21 (20)	18 (17)	14 (13)	9 (8)	4 (4)	2 (2)	0
Cutoff>=0.9	18 (17)	13 (12)	9 (8)	6 (6)	1 (1)	0	0
Cutoff>=0.95	14 (13)	10 (9)	4 (3)	1 (1)	0	0	0
Cutoff>=0.99	10 (9)	4 (4)	0	0	0	0	0
**Class3**	Number of variants **(**and associated genes**)** passing filter: **[**rank-score>=Cutoff in at least **N** of **7** meta-tools**]**						
Total: 752 (849)	**N=1**	**N=2**	**N=3**	**N=4**	**N=5**	**N=6**	**N=7**
Cutoff>=0.8	258 (224)	162 (143)	104 (98)	64 (61)	31 (31)	**13 (13)**	3 (3)
Cutoff>=0.85	210 (186)	111 (101)	63 (61)	34 (34)	13 (13)	4 (4)	1 (1)
Cutoff>=0.9	143 (133)	68 (67)	35 (36)	8 (8)	2 (2)	0	0
Cutoff>=0.95	85 (82)	29 (30)	11 (12)	1 (1)	0	0	0
Cutoff>=0.99	**14 (14)**	1 (1)	0	0	0	0	0
**ClassUNKNOWN**	Number of variants **(**and associated genes**)** passing filter: **[**rank-score>=Cutoff in at least **N** of **7** meta-tools**]**						
Total: 20541 (14043)	**N=1**	**N=2**	**N=3**	**N=4**	**N=5**	**N=6**	**N=7**
Cutoff>=0.8	2547 (2120)	1503 (1289)	886 (773)	520 (480)	305 (291)	151 (151)	**72 (70)**
Cutoff>=0.85	2037 (1707)	1057 (917)	561 (510)	307 (294)	158 (158)	85 (87)	26 (26)
Cutoff>=0.9	1447 (1235)	636 (570)	293 (276)	142 (141)	67 (69)	34 (35)	6 (6)
Cutoff>=0.95	769 (688)	240 (225)	89 (89)	27 (27)	15 (15)	1 (1)	0
Cutoff>=0.99	165 (155)	**20 (20)**	1 (1)	0	0	0	0
